# Assessing the Evolution and Influence of Medical Open Databases on Biomedical Research and Health Care Innovation: A 25-Year Perspective With a Focus on Privacy and Privacy-Enhancing Technologies

**DOI:** 10.2196/58954

**Published:** 2026-01-30

**Authors:** Albert Yang, Mei-Lien Pan, Henry Horng-Shing Lu, Chung-Yueh Lien, Da-Wei Wang, Chih-Hsiung Chen, Der-Cherng Tarng, Dau-Ming Niu, Shih-Hwa Chiou, Chun-Ying Wu, Ying - Chou Sun, Shih-Ann Chen, Shuu-Jiun Wang, Wayne Huey-Herng Sheu, Chi-Hung Lin

**Affiliations:** 1 Digital Medicine and Smart Healthcare Research Center, National Yang Ming Chiao Tung University, No. 155 Sec. 2 Linong St., Beitou Dist, Taipei City, 112, Taiwan, 886228267995; 2 School of Medicine, National Yang Ming Chiao Tung University, Taipei, Taiwan; 3 Department of Medical Research, Taipei Veterans General Hospital, Taipei, Taiwan; 4 Institute of Hospital and Health Care Administration, National Yang Ming Chiao Tung University, Taipei, Taiwan; 5 Institute of Statistics, National Yang Ming Chiao Tung University, Hsinchu, Taiwan; 6 Biomedical Artificial Intelligence Academy, Kaohsiung Medical University, Kaohsiung, Taiwan; 7 Department of Artificial Intelligence in Medicine, Kaohsiung Medical University, Kaohsiung, Taiwan; 8 Department of Medical Research, Kaohsiung Medical University Hospital, Kaohsiung, Taiwan; 9 Department of Statistics and Data Science, Cornell University, Ithaca, NY, United States; 10 Department of Information Management, National Taipei University of Nursing and Health Sciences, Taipei, Taiwan; 11 Institute of Information Science, Academia Sinica, Taipei, Taiwan; 12 Institute of Technology Law, National Yang Ming Chiao Tung University, Taipei, Taiwan; 13 Department of Medicine, Taipei Veterans General Hospital, Taipei, Taiwan; 14 Institute of Clinical Medicine, National Yang Ming Chiao Tung University, Taipei, Taiwan; 15 Department and Institute of Physiology, National Yang Ming Chiao Tung University, Taipei, Taiwan; 16 Department of Pediatrics, Taipei Veterans General Hospital, Taipei, Taiwan; 17 Institute of Pharmacology, College of Medicine, National Yang Ming Chiao Tung University, Taipei, Taiwan; 18 Institute of Biomedical Informatics, National Yang Ming Chiao Tung University, Taipei, Taiwan; 19 Health Innovation Center, National Yang Ming Chiao Tung University, Taipei, Taiwan; 20 Department of Radiology, Taipei Veterans General Hospital, Taipei, Taiwan; 21 Department of Cardiovascular Center and Medical Research, Taichung Veterans General Hospital, Taichung, Taiwan; 22 Department of Neurology, Neurological Institute, Taipei Veterans General Hospital, Taipei, Taiwan; 23 Brain Research Center, National Yang Ming Chiao Tung University, Taipei, Taiwan; 24 Institute of Molecular and Genomic Medicine, National Health Research Institutes, Taipei, Taiwan; 25 Department of Biological Science & Technology, National Chiao Tung University, Taipei, Taiwan

**Keywords:** medical open databases, artificial intelligence, privacy-enhancing technologies, federated learning, dynamic consent systems

## Abstract

The integration of medical open databases with artificial intelligence (AI) technologies marks a transformative era in biomedical research and health care innovation. Over the past 25 years, initiatives like PhysioNet have revolutionized data access, fostering unprecedented levels of collaboration and accelerating medical discoveries. This rise of medical open databases presents challenges, particularly in harmonizing research enablement with patient confidentiality. In response, privacy laws such as the Health Insurance Portability and Accountability Act have been established, and privacy-enhancing technologies have been adopted to maintain this delicate balance. Privacy-enhancing technologies, including differential privacy, secure multiparty computation, and notably, federated learning (FL), have become instrumental in safeguarding personal health information. FL, in particular, represents a significant advancement by enabling the development and training of AI models on decentralized data. In Taiwan, significant strides have been made in aligning with these global data-sharing and privacy standards. We have actively promoted the sharing of medical data through the development of dynamic consent systems. These systems enable individuals to control and adjust their data-sharing preferences, ensuring transparency and continuity of consent in the ever-evolving landscape of digital health. Despite the challenges associated with privacy protections, the benefits, including improved diagnostics and treatment, are substantial. The availability of open databases has notably accelerated AI research, leading to significant advancements in medical diagnostics and treatments. As the landscape of health care research continues to evolve with open science and FL, the role of medical open databases remains crucial in shaping the future of medicine, promising enhanced patient outcomes and fostering a global research community committed to ethical integrity and privacy.

## Introduction

The emergence of medical open databases, coupled with advances in artificial intelligence (AI), heralds a significant change in biomedical research and health care innovation, facilitating an era of enhanced accessibility and data sharing [1-3]. This movement toward open data science, augmented by AI technologies, enables researchers worldwide to access a wealth of data, including physiological signals [4,5], genomic [6], and health care information [7], and, most prominently, large-scale medical imaging archives. While this review covers the broad spectrum of medical data, the impact of open imaging databases has been particularly transformative for the application of AI. This movement toward open data science fosters collaboration and speeds up the pace of medical discoveries.

AI’s role in analyzing vast datasets has been instrumental in uncovering patterns and insights that would be impossible for humans to detect unaided, leading to breakthroughs in understanding diseases and patient care. Initiatives like annual challenges and shared toolboxes have spurred the development of novel algorithms and techniques, leveraging AI to address complex biomedical challenges and advance medical diagnostics and treatments. This synergy between open medical databases and AI is transforming the landscape of health care, promising a future of more accurate, efficient, and personalized medicine.

Simultaneously, this rise in open data repositories brings to the forefront crucial privacy concerns [6,8]. The necessity to balance the imperative of research enablement with the protection of patient confidentiality has never been more pronounced. In this context, laws such as the Health Insurance Portability and Accountability Act (HIPAA) play a pivotal role in shaping the landscape of data deidentification and anonymization processes, ensuring that shared data comply with strict privacy standards [9]. Moreover, the introduction of privacy-enhancing technologies (PETs), such as differential privacy [10], synthetic data [11], homomorphic encryption [12], secure multiparty computation [13], and federated learning [14], represents a proactive approach to safeguarding personal health information. These technologies provide the means to conduct meaningful research while upholding the principles of data privacy and security.

In a country like Taiwan, strides in medical data sharing suggest the global shift toward interconnected health systems, highlighting both advancements and ongoing challenges in securing patient data. The implementation of dynamic consent frameworks reflects a growing recognition of the need for more flexible approaches to data privacy, particularly in an era of personalized medicine and digital health records [15]. As the landscape of medical research evolves with these developments, the interplay of data sharing, privacy, and technology continues to reshape the boundaries of what is possible in health care innovation, marking a critical junction in the journey toward more open, collaborative, and ethically responsible research environments.

This review aims to provide a 25-year perspective on the evolution of medical open databases, tracing their impact on biomedical research and health care innovation, and to examine how emerging PETs and data-governance frameworks, including dynamic consent systems, shape the ethical, technical, and collaborative landscape of digital medicine worldwide.

## The Rise of Medical Open Database

The rise of medical open databases represents a transformative shift in the landscape of biomedical research and health care innovation. Among the pioneers in this movement is PhysioNet. Established in 1999, PhysioNet is a pioneering open database that provides free access to a wide range of physiologic signals and related open-source software for research in medicine, physiology, and biomedical engineering [4,16]. It was initiated by a collaborative project involving researchers from Boston’s Beth Israel Deaconess Medical Center/Harvard Medical School, Boston University, McGill University, and Massachusetts Institute of Technology [4,17]. The database contains a diverse collection of physiological datasets, including those related to cardiovascular and other complex biomedical signals [4,18]. PhysioNet has had a significant impact on the development of medical open databases, serving as a model for the establishment of similar resources. It has also played a key role in promoting the dissemination and exchange of medical resources.

A significant contribution of PhysioNet to the scientific community is its annual PhysioNet Challenge, which has markedly influenced the field by promoting innovation and collaboration among researchers and clinicians. These challenges stimulate the creation of novel algorithms and methods aimed at solving complex biomedical problems, thus expanding the limits of what can be achieved in medical data analysis and application. For instance, the challenges have catalyzed the development of innovative algorithms capable of detecting obstructive sleep apnea from electrocardiograms [19], illustrating the practical impact of these competitions on advancing medical diagnostics and treatment strategies [20].

**Figure 1.**
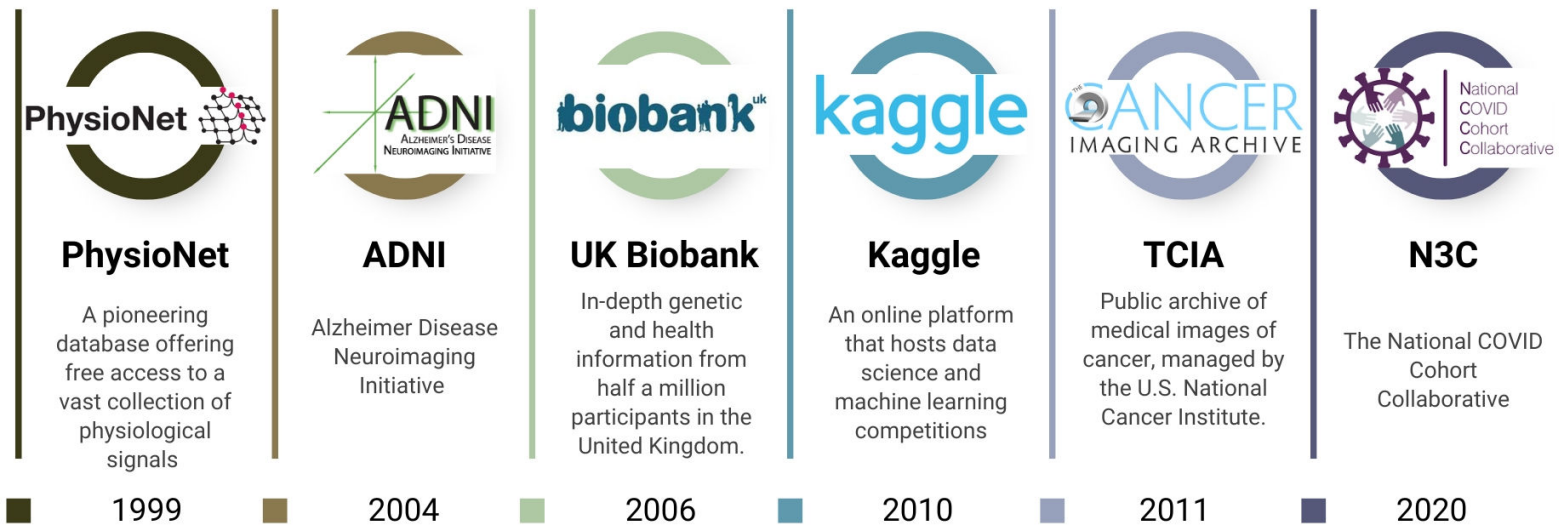
Historical development of major medical open databases and data-sharing platforms over the past 25 years.

Since the success of PhysioNet, numerous other medical open databases have emerged globally, fostering a more cooperative and transparent research atmosphere (Figure 1). The impact of these large-scale databases on biomedical discovery is profound. One notable example is the UK Biobank [21], launched in 2006, which provides a vast repository of genetic and health information from half a million UK participants. This database has become an essential tool for unraveling the complex interplay between genetics, lifestyle, and disease, thereby enhancing our understanding of the factors influencing human health [22-25]. By leveraging large neuroimaging cohorts such as Alzheimer Disease Neuroimaging Initiative and the UK Biobank, researchers have developed AI-based models that generate an Alzheimer disease risk score from structural magnetic resonance imaging (MRI), enabling the identification of prediagnostic populations suitable for early intervention and preventive trials [26]. In cardiovascular research, analysis of the UK Biobank’s genetic and imaging data has enabled the development of NeuralCVD, a neural network–based risk model that integrates polygenic and clinical predictors to estimate the 10-year risk of major adverse cardiac events, improving risk discrimination and reclassification beyond established clinical scores and Cox models, and highlighting the added predictive value of genetic predisposition in early prevention [27]. Similarly, the Cancer Imaging Archive, inaugurated in 2011 in the United States, offers a dedicated platform for the cancer research community, enabling access to a comprehensive array of imaging datasets [28].

Beyond these examples, the ecosystem of medical open databases has diversified into numerous specialized domains. For instance, OpenNeuro provides a vast repository for neuroimaging data, particularly functional magnetic resonance imaging, electroencephalogram, and magnetoencephalography, supporting reproducible brain research [29]. The Neuroimaging Informatics Tools and Resources Clearinghouse offers a rich collection of imaging and data-processing tools [30]. Similarly, the National Database for Autism Research [31] and the Federal Interagency Traumatic Brain Injury Research informatics system [32] provide deeply phenotyped datasets crucial for research in their respective fields. These platforms underscore the field’s shift toward creating specialized, high-quality resources to tackle specific biomedical questions. They display how shared resources can drive forward innovation and improve patient care worldwide, illustrating the critical role of collaborative environments in the advancement of health care research and application.

Additionally, Kaggle, an online platform for data science and machine learning (ML) competitions, has emerged as a pivotal player in the field of ML analysis and data sharing [33]. Launched in 2010, Kaggle facilitates collaboration and competition among data scientists and researchers by hosting challenges in various domains, including health care. These competitions often involve complex medical datasets, encouraging participants to develop innovative solutions and algorithms for disease prediction, medical imaging analysis [34], and other health-related issues. Kaggle has not only democratized access to large medical datasets but has also fostered a global community where knowledge and techniques are openly shared. This environment has led to significant breakthroughs and advancements in medical research and analytics, further suggesting the importance of open data and collaborative problem-solving in improving health care outcomes and accelerating medical innovation.

## Balancing Privacy and the Need for Medical Open Databases

The success of open medical databases such as PhysioNet poses the challenge of balancing patient confidentiality with research enablement on open platforms [35,36]. Research datasets in health care often contain protected health information (PHI), and the process of removing this information, a process known as deidentification or anonymization, can be challenging and prone to errors [37]. Despite the use of these datasets, the need for deidentification introduces a significant barrier to data sharing due to the effort and cost involved.

The HIPAA, established in the United States in 1996, plays a vital role in safeguarding patients’ medical information. In response to the HIPAA mandate, U.S. Department of Health and Human Services published a final regulation in the form of the privacy rule in December 2000, which became effective on April 14, 2001. Central to this rule is the designation of 18 specific categories of PHI that, if disclosed, could be used to identify an individual (Textbox 1). These categories encompass a broad spectrum of personal data, including, but not limited to, names, geographic details smaller than a state, various identifiers like social security numbers, medical record numbers, and contact information, as well as certain biometric and photographic images [38].

Textbox 1. **Eighteen categories of protected health information.**

- Names
- All geographic subdivisions smaller than a State
- All elements of dates (except year) for dates directly related to an individual
- Telephone numbers
- Fax numbers
- Electronic mail addresses
- Social security numbers
- Medical record numbers
- Health plan beneficiary numbers
- Account numbers
- Certificate/license numbers
- Vehicle identifiers and serial numbers
- Device identifiers and serial numbers
- Web URLs
- IP address numbers
- Biometric identifiers, including finger and voice prints
- Full face photographic images and any comparable images
- Any other unique identifying number, characteristic, or code

Additionally, HIPAA mandates that covered entities must ensure they do not possess knowledge that the remaining information could be used, whether alone or in conjunction with other data, to identify the subject. By strictly adhering to these guidelines, entities can share deidentified health information for broader uses, such as public health and research, without infringing on individual privacy rights, thus striking a balance between privacy protection and the beneficial use of health data [39]. Despite these efforts, the tension between the promise of big data and patient privacy in health care research remains a challenge [40].

PhysioNet is a pioneer in medical public databases, ensuring that the datasets it provides do not compromise individual privacy. This involves ensuring that any data shared does not contain PHI or has been sufficiently anonymized to prevent the identification of individuals. The challenges posed by the HIPAA privacy rule are not insignificant; they include the need for informed consent from data subjects and potential limitations on access to health information that can hinder clinical research [41,42]. Furthermore, the rule’s interaction with other regulations, like the common rule, adds complexity to privacy concerns in research, leading to inconsistencies and additional burdens for researchers.

Despite these challenges, the privacy rule does allow for certain disclosures without patient authorization, particularly for public health purposes. This is intended to facilitate the use of medical data in important public health endeavors without undermining individual privacy protections [43]. The balance sought by the HIPAA privacy rule between protecting privacy and facilitating research is a critical aspect of its implementation, particularly in the context of medical open databases. By navigating these regulations successfully, repositories can contribute to the advancement of medical research while ensuring compliance with privacy standards [44].

The emergence of medical databases, such as the PhysioNet, UK Biobank, and the Cancer Imaging Archive, has significantly advanced collaborative research in health care [45,46]. These databases have the potential to transform cancer research and improve patient outcomes [45]. However, the collection, linking, and use of data in biomedical research raise ethical concerns, particularly regarding privacy and security [36,47,48]. Despite these concerns, the benefits of open data in health care, including improved diagnostics and treatment, are substantial [48]. The push for data sharing in cancer trials by pharmaceutical companies further underscores the importance of open medical databases in driving innovation and improving patient care [49].

## Privacy Enhancing Technology

### Overview

A range of studies have been conducted to explore the increasing frequency and impact of health care data breaches, highlighting the rising number of incidents and their detrimental effects on patient privacy and health care providers [50-53]. These breaches are often caused by a combination of technical, organizational, and human factors [50-52]. Human vulnerabilities, such as lack of awareness and training, play a significant role in these breaches [51]. The use of the Swiss Cheese Model can help assess vulnerabilities and risks [50]. Cloud computing breaches are a particular concern, highlighting the need for digital forensic readiness [54]. Hacking and unauthorized internal disclosures are the most prevalent forms of attack [53]. Further studies may examine specific cases and the implications for digital forensic readiness, emphasizing the importance of adhering to regulations.

Below, we reviewed several PETs and their applications in enhancing data privacy and security in health care settings (Table 1). PETs, such as encryption, anonymization techniques, and secure multiparty computation, offer powerful mechanisms to protect sensitive health data. Implementing these technologies, alongside robust privacy policies and employee training, can significantly reduce the likelihood of data breaches and bolster the trust between patients and health care providers.

**Table 1.** Summary of privacy-enhancing technologies.

Technologies	Core principle	Advantages	Challenges and trade-offs
Differential privacy	Adds calibrated statistical noise to query results to make it impossible to determine if an individual’s data were included.	Provides strong and mathematically provable privacy guarantees.	Inherent trade-off between privacy and data use; high privacy can reduce analytical accuracy.
Synthetic data	Creates an artificial dataset that mimics the statistical properties of the original data without containing real patient information.	High use for model training; no real patient data are shared, eliminating reidentification risk.	Can be difficult to generate high-fidelity data that captures all complex correlations; potential for model bias.
Homomorphic encryption	Allows computations to be performed directly on encrypted data without decrypting it first.	Offers extremely strong security, as the raw data are never exposed.	High computational overhead; currently too slow for many complex ML^a^ tasks.
Secure multiparty computation	Enables multiple parties to jointly compute a function over their inputs while keeping those inputs private.	Allows for collaborative analysis without a central data repository; no single party sees another’s data.	High communication overhead between parties; can be complex to set up and scale.
Federated learning	Trains a central AI^b^ model across decentralized devices or servers holding local data samples, without exchanging the data itself.	Keeps raw data local, enhancing privacy and data sovereignty.	Vulnerable to model poisoning/inversion attacks; performance can degrade with heterogeneous data.

a ML: machine learning.

b AI: artificial intelligence.

### Differential Privacy

Differential privacy, a method for protecting individual privacy in data analysis, has been increasingly applied in the health care sector. It involves adding noise to the data to prevent reidentification of individuals. This approach has been used in various areas of health research, including genomics, neuroimaging, and health surveillance [55]. However, there are challenges in its practical application, such as the theoretical nature of the privacy parameter epsilon [56]. To address these challenges, researchers have proposed differentially private data release strategies and noise mechanisms, such as the Laplace and exponential mechanisms [57]. However, a key challenge is the inherent trade-off between privacy and data use; increasing the amount of statistical noise to protect privacy can reduce the accuracy of analytical outcomes.

The application of differential privacy in medical questionnaires has also been explored, with the randomized response mechanism showing promise in improving privacy while retaining data use [58]. Furthermore, the use of differential privacy in geospatial analyses of standardized health care data has been demonstrated, with the development of geodatabase functions for privacy-aware analysis [59]. Finally, the combination of differential privacy and decision tree approach has been proposed for data publishing, and the differentially private mini-batch gradient descent algorithm for model publishing of medical data [60].

### Synthetic Data

Synthetic data, generated through simulators, is increasingly used in health care to address the challenges of data availability and privacy [61]. PETs, such as differential privacy, are combined with synthetic data generators to create private synthetic data, preserving statistical properties while ensuring privacy [62]. These technologies have been applied in various use cases, including clinical risk prediction [63] and medical research [64]. However, the evaluation of synthetic data’s privacy and use metrics remains a challenge, with a lack of consensus on standard approaches [65]. Despite these challenges, the potential of synthetic data in preserving data use and patient privacy in electronic health care data is being explored [66].

### Homomorphic Encryption

Homomorphic encryption, a powerful tool for preserving privacy in medical data, allows for computations to be performed on encrypted data without the need for decryption. It has been successfully applied in various medical data scenarios, including ML models for classification and training, secure genomic algorithms, and predictive analysis tasks [67-69]. For example, it has been used to securely manage personal health metrics data, process medical images [70,71], and enable secure medical computation [72]. The use of homomorphic encryption in these applications ensures that sensitive medical data remains private and secure. Despite its power, the primary limitation of homomorphic encryption is its significant computational overhead, which can make it slow and resource-intensive for complex computations on large datasets.

### Secure Multiparty Computation

Secure multiparty computation is a cryptographic technique that enables data analytics without sharing the underlying data, making it a valuable tool for preserving privacy in medical data analysis [73]. It has been applied in various health care scenarios, including collaborative systems [74], statistical analysis of health data [75], and electronic medical record (EMR) data [75]. Secure multiparty computation has also been used in health care internet of things systems to handle privacy issues [76], prevent data disclosure in sensor networks [77], and enable the reuse of distributed electronic health data [75]. Furthermore, it has been applied to enable privacy-preserving query processing on EMRs [78]. Notably, secure multiparty computation has enabled research on highly sensitive data (such as HIV, rare diseases, and population genomics) that would otherwise be inaccessible due to privacy concerns.

### Federated Learning

Federated learning (FL), a decentralized ML approach, is increasingly being applied in the medical field due to the sensitive and fragmented nature of health care data [14,79]. It allows for the collaborative development of ML models without sharing raw data, thus preserving privacy [80,81]. This approach has been used in various medical domains, including oncology and radiology, for tasks such as image analysis and disease prediction [81,82]. However, there are challenges to be addressed, such as data homogeneity and transparency [81]. Furthermore, FL can be vulnerable to security risks, such as model inversion attacks that attempt to reconstruct training data from the shared model updates, and require careful design to ensure robustness. Despite these challenges, FL shows promise in improving the efficiency and privacy of medical data processing [83-85].

To address the challenge of data heterogeneity in FL, we have proposed the Dynamically Synthetic Images for Federated Learning method, significantly improving the conventional FL framework by integrating local information from local multiple institutions with heterogeneous data types [86]. The core principle of its implementation involves a dynamic process where, at the start of each training round, a client’s local data are evaluated by the current global model to identify misclassified images. Using a synthetic minority oversampling technique, the system generates new, synthetic images based on these misclassified cases, which are then added to the local training set to compel the model to focus on features it previously failed to learn. In terms of effectiveness, experimental results demonstrated that Dynamically Synthetic Images for Federated Learning-based models achieve higher accuracy than conventional FL approaches and that their performance can be comparable to that of traditional centralized learning, proving especially beneficial for institutions with smaller or more heterogeneous datasets [86].

## Taiwan Medical AI and Data Portal and Dynamic Consents System

Taiwan has made significant strides in medical data sharing, particularly in the areas of privacy protection and electronic health records exchange. The country’s comprehensive embedded integrated circuit-based health insurance card system, implemented by the Bureau of National Health Insurance, Taiwan, allows for the secure sharing of health information [87]. The use of blockchain technology has been proposed as a means to further enhance the security and privacy of medical data sharing [88,89]. The Taiwan Electronic Medical Record Template and the National Electronic Medical Record Exchange System have been developed to facilitate the exchange of EMRs [90,91]. However, concerns about unauthorized access and secondary use of EMRs persist, particularly among highly educated individuals [92]. The country has also established guidelines for the security and privacy protection of health information, drawing on international best practices [93].

**Figure 2.**
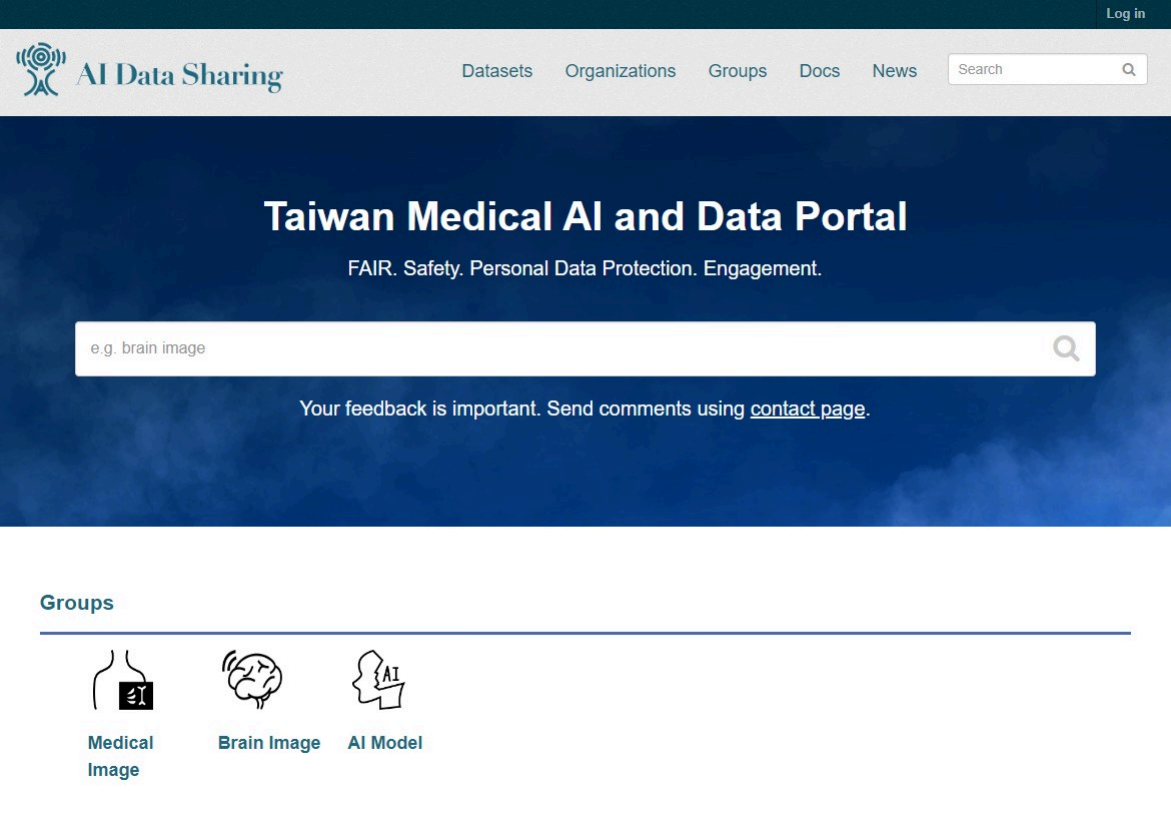
Taiwan medical artificial intelligence and data portal. AI: artificial intelligence.

In the past 4 years, funded by the National Science and Technology Council of Taiwan, we have assembled teams from National Yang Ming Chiao Tung University, Taipei Veterans General Hospital, Academia Sinica, and National Taipei University of Nursing and Health Sciences to form a data repository task force known as the Smart Medical AI and Repository Taskforce Center. We launched a medical AI and data-sharing platform aimed at advancing the field of medical AI research in October 2023 [94]. This platform not only provides the public and researchers with access to a multitude of shared datasets but also ensures a meticulous evaluation process (Figure 2). Researchers can apply for access to the data by providing an abstract of their research proposal. Dataset managers assess applications based on their intended use, specific needs, and detailed research plans.

Currently, seventeen datasets have been released on the platform, covering neuropsychiatric disorders, brain tumors, ophthalmic diseases, musculoskeletal disorders, and cardiopulmonary diseases. All datasets have undergone deidentification and delinking processes and include annotated information to facilitate AI training and validation. Specifically, our data-sharing platform includes: MRI images of vestibular schwannoma; computed tomography (CT) images of intracerebral hemorrhage; brain Fluorodeoxyglucose-Positron Emission Tomography/Magnetic Resonance Imaging images for dementia diagnosis; primary brain tumor MRI datasets, including meningioma, glioma, and pituitary adenoma; MRI data of brain metastases, which represent the largest collection nationwide; hand and foot X-rays of rheumatoid arthritis; X-rays of compression fractures; spinal X-rays of ankylosing spondylitis; chest CT images and clinical data of atrial fibrillation patients; chest X-rays for lung cancer screening; annotated preoperative liver CT images; neck lymph node CT images with postoperative pathology results; the Taiwan Aging and Mental Illness Cohort brain imaging database; the dementia molecular imaging database; fundus image datasets for glaucoma; and fundus image datasets of polypoidal choroidal vasculopathy.

The data sharing platform is built on a comprehensive architecture designed to support AI research by integrating 3 core systems: a CKAN-based sharing platform for dataset management, a data application system, and a dynamic authorization consent platform for patient privacy. Specific features include a robust user authentication and authorization mechanism, allowing dataset managers to grant access to specific users or collaborators. The platform ensures data integrity and ethical compliance through a multistep deidentification process for all medical images and by linking to the dynamic consent system (for sensitive clinical data), which allows patients to manage their data sharing preferences in real-time. To use the database, researchers first search for datasets on the platform, then apply for access through a formal registration and review process. Once approved and authorized by the dataset manager, users can obtain a login key to programmatically access the data through standardized protocols, such as DICOMweb, ensuring a secure, convenient, and interoperable environment for third-party AI applications.

This effort aims to advance research across 7 crucial clinical areas that greatly benefit from AI technology, including heart disease, neurological disorders, mental illness, diabetes, cancer, genetic predispositions to complex diseases, and medical imaging. Moreover, the platform underpins collaboration between distinct teams specializing in AI methodology, science and law, and data governance, jointly fostering a robust data governance framework that emphasizes FL, cloud-based AI solutions, and trusted AI practices. Importantly, the system is designed to streamline the research process while maintaining a focus on ethical standards and participant privacy. In line with this, the platform incorporates a dynamic informed consent mechanism, especially for datasets that are anonymized but cannot be completely separated from their sources. This approach ensures that participants’ privacy is safeguarded while also enabling their informed and ongoing consent, reflecting our commitment to ethical research practices and the dynamic nature of consent in medical studies.

Dynamic informed consent, a concept that has been explored in various contexts, is a personalized, digital communication interface that allows participants to manage their consent preferences [95]. It has been proposed as a solution to improve patient confidence and trust in the use of electronic patient records in medical research [96]. In the context of privacy-aware pervasive health and well-being, dynamic consent enables granular data consent and management [97]. It has also been suggested as a potential solution to challenges in modern biomedical research, including participant recruitment, informed consent, and consent management [98]. The use of blockchain technology has been proposed to enhance the privacy-preserving aspects of dynamic consent in genomic data sharing [99]. The concept of dynamic informed consent has been further explored in the context of personalized medicine, emphasizing the need for a more dynamic and enriched consent model [100].

**Figure 3.**
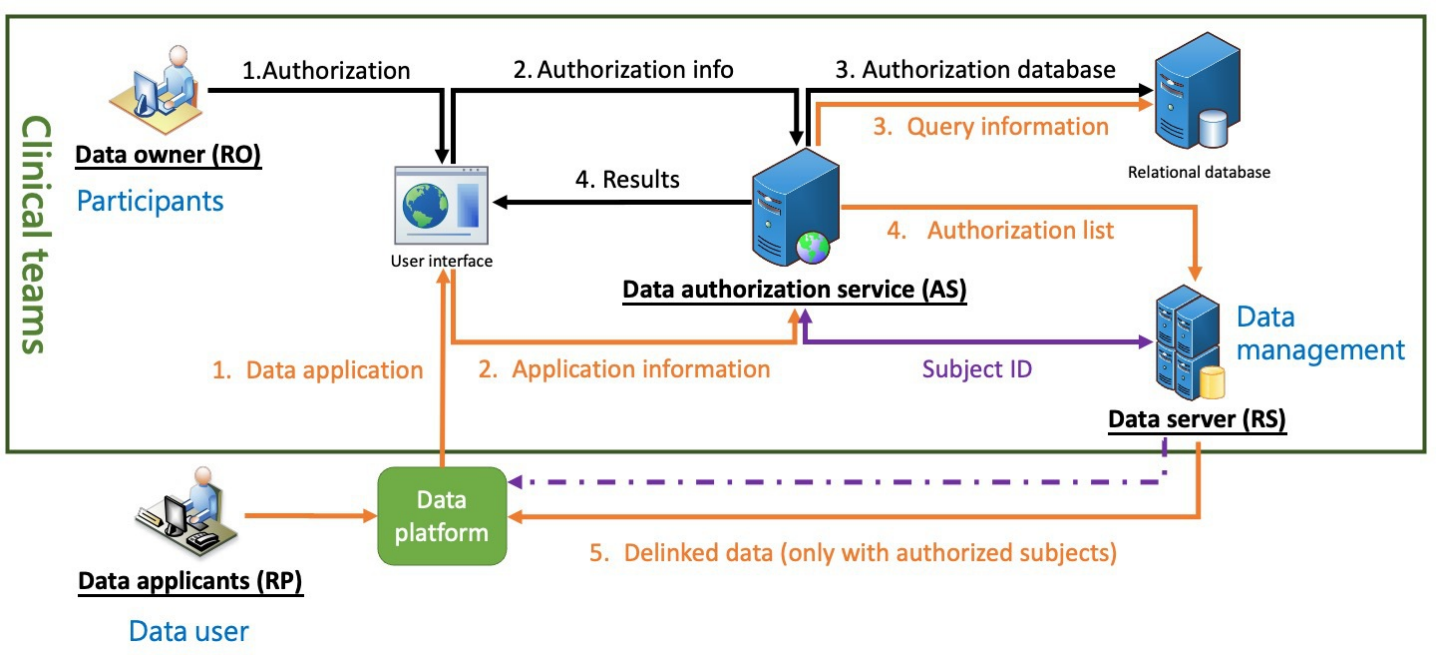
Health data authorization service platform. This diagram depicts the architecture of a medical data-sharing platform that integrates dynamic consent for secure and transparent data sharing. The system centers on 4 key roles: the resource owner (RO), who owns the data; the resource server (RS), where medical data are stored and managed; the requesting party (RP), typically researchers seeking data access; and the authorization server (AS), the core component facilitating connections among the roles. This framework ensures data access is based on explicit consent from the data owner, allowing real-time adjustments to consent settings for different data types and uses, such as academic research. The platform’s primary tasks are to establish individual preferences, maintain consent history, and ensure trust and transparency between data owners and users, thereby advancing responsible data science development.

To enhance the privacy of participants contributing to the data in our platform and increase participants’ engagement, we have developed a dynamic informed consent system, named the Health Data Authorization Service Platform (Figure 3). This collaborative effort involves our data governance, humanities, science and law, and clinical teams, aiming to facilitate scalable, dynamic consent operations suitable for complex data environments. The platform supports flexible data governance, allowing data owners to dynamically express their consent preferences, thereby making dynamic consent practical and sustainable.

The platform’s architecture encompasses 4 key roles: the resource owner, usually the participants, who owns the data; the resource server, which stores and manages medical data; the requesting party, typically researchers seeking data access; and the authorization server, the backbone of our dynamic consent system, connecting the 3 roles. This system ensures that data are accessed only with the owner’s express consent, respecting their preferences and enhancing data use transparency. Resource owners can modify their consent settings at any time, reflecting changes in their willingness to share different data types, like EMRs or medical imaging, for specific purposes such as academic research. This flexibility ensures that data use aligns with the owners’ current preferences. The platform seamlessly integrates with our shared data framework, maintaining each citizen’s consent history and enabling swift updates to consent forms as needed. By streamlining the consent process and ensuring data are shared according to owner permissions, our platform respects individual preferences while promoting responsible data science development. It exemplifies a forward-thinking approach to data governance, enabling real-time adjustments in consent and fostering a culture of trust and transparency between data owners and users.

These initiatives in Taiwan can be understood within the global context of evolving data privacy regulations. Unlike the “one-time, broad consent” model often used in US-based research under the Common Rule, Taiwan’s move toward dynamic consent aligns more closely with the principles of the European Union’s General Data Protection Regulation [101]. The General Data Protection Regulation mandates that consent must be specific, informed, and easily revocable. The dynamic consent system builds on this by providing a technological interface for participants to manage their preferences granularly and continuously, representing a best-practice approach to balancing research needs with individual autonomy and privacy rights.

## Acceleration of Medical AI Research Through Open Databases

The advent of medical open databases has significantly accelerated the field of medical AI research, fostering an environment of innovation and rapid development [102]. By providing researchers with access to vast amounts of health-related data, these databases have become a cornerstone for advancements in predictive analytics, diagnostic algorithms, and personalized medicine.

One of the most notable contributions of open medical databases to AI research is the democratization of data [103]. Historically, the scarcity and inaccessibility of medical data posed substantial hurdles to AI development. However, platforms like PhysioNet, established in 1999, have bridged this gap by offering a plethora of datasets ranging from physiological signals to clinical outcomes [104]. However, challenges remain, including the need for large datasets and the lack of external validation in perioperative medicine [105]. The use of open science approaches, including data liberation and crowdsourcing, can help address these challenges [106]. The integration of networked medical devices and clinical repositories based on open standards can further enhance AI research in high-acuity medical environments [107]. This enhanced availability allows researchers from diverse backgrounds and institutions to engage in health care innovation, leveling the playing field and stimulating a surge in AI-based solutions.

The availability of open databases has catalyzed the application of diverse AI methodologies to complex medical problems. Deep learning, particularly convolutional neural networks, has achieved state-of-the-art performance by leveraging large-scale imaging datasets; for example, researchers have trained convolutional neural networks on millions of images from The Cancer Imaging Archive to develop algorithms capable of detecting and classifying tumors in radiological scans with accuracy comparable to human experts [108]. For structured data such as the genetic and clinical information in the UK Biobank, traditional ML models like random forests and gradient boosting have been widely used, excelling at identifying complex patterns to predict disease risk, including the calculation of polygenic risk scores for coronary artery disease based on thousands of genetic variants [109]. In addition, natural language processing techniques have been applied to large repositories of unstructured clinical notes, such as those in the Medical Information Mart for Intensive Care version IV (MIMIC-IV) database (part of PhysioNet), to extract critical information on symptoms, treatments, and outcomes, thereby enabling large-scale retrospective studies that were previously infeasible [110].

Open medical databases encompass a wide variety of data types, including EMRs, imaging, genomic sequences, and more. This diversity enables AI researchers to explore multifaceted health care questions, from predicting disease trajectories to optimizing treatment plans. Moreover, the rich, varied datasets facilitate the training of more robust and generalizable AI models, capable of addressing complex medical scenarios across different populations and settings. The shared nature of open databases fosters collaboration across the global research community [111]. Through platforms that offer shared data, researchers can combine their expertise to tackle larger and more complex problems than they could individually. This collaborative approach has led to significant breakthroughs in AI, such as algorithms that can detect diseases from images with accuracy rivaling that of trained professionals [112,113].

Open databases also streamline the validation and implementation phases of AI development [114]. Access to diverse datasets enables researchers to rigorously test their algorithms under various conditions and patient demographics, ensuring their reliability and effectiveness. The expansion of these databases has significantly propelled medical AI research forward, marking a new phase of health care innovation with faster discoveries, collaborative efforts, and a commitment to ethical data use. As the field evolves, the role of open databases in shaping the future of medicine remains pivotal.

## Conclusions

In conclusion, the evolution toward medical open databases, exemplified by the inception of platforms like PhysioNet in 1999 and their progression over the past 25 years, alongside the integration of PETs, marks a significant milestone in the domain of biomedical research and health care innovation. This journey not only fosters an unprecedented level of collaboration and accessibility but also emphasizes the crucial need to address privacy concerns and ethical considerations diligently. The ongoing efforts to balance data sharing with individual privacy protection are underscored by the adaptation of legal frameworks and the implementation of cryptographic and data management solutions. The introduction and growth of medical open databases have been pivotal, providing a wealth of data that has propelled research and innovation while highlighting the challenges and responsibilities of managing sensitive information. Specifically, the availability of open medical databases has significantly accelerated AI research, leading to breakthroughs in disease prediction, diagnostics, and personalized medicine. As we continue to explore the vast potential of open science and FL, the landscape of health care research is on the brink of remarkable transformations. These advancements promise enhanced patient outcomes, faster medical discoveries, and a more inclusive global research community, all achieved by adhering to the highest standards of privacy and ethical integrity.

## Abbreviations

AI: artificial intelligence
CT: computed tomography
EMR: electronic medical record
FL: federated learning
HIPAA: Health Insurance Portability and Accountability Act
ML: machine learning
MRI: magnetic resonance imaging
PET: privacy-enhancing technology
PHI: protected health information
